# Correcting PVS1 overestimation: clinical insights into rescue transcripts and variant reclassification in 4 prenatal cases

**DOI:** 10.3389/fmed.2026.1799731

**Published:** 2026-05-13

**Authors:** Xiangyou Leng, Jin Wang, Danying Yi, Xiaoyan Zhu, Shengfang Qin, Xinying Wang, Rui Tang, Xueyan Wang

**Affiliations:** 1 Department of Medical Genetics and Prenatal Diagnosis, Sichuan Provincial Women's and Children's Hospital, The Affiliated Women's and Children's Hospital of Chengdu Medical College, Chengdu, China; 2 Department of Clinical Laboratory, Sichuan Provincial Women's and Children's Hospital, The Affiliated Women's and Children's Hospital of Chengdu Medical College, Chengdu, China; 3 Department of Medical Genetics, West China Medical School, Sichuan University, Chengdu, China

**Keywords:** ACMG, AlphaFold3, ES, PVS1, rescue transcript

## Abstract

**Background:**

As NGS has become more widely used in the diagnosis of rare diseases, an increasing number of variants of uncertain significance (VUS) have been detected, and their interpretation remains challenging. In particular,when a LOF variant in a non-conserved exon of the MANE transcript is detected by ES, yet the individual carrying this variant exhibits no clinical manifestations. In this context, the ACMG Splicing Subgroup introduced the term 'rescue transcripts' to guide the interpretation of LOF variants located within non-conserved exons.

**Methods:**

In this study, the properties of four different variants were characterised using in silico predictions, protein structural analysis, population frequency data, and clinical observations.

**Results:**

Three variants (*AMMECR1*:c.220C>T; *GLI2*:c.162_163delCT; *COL11A1*:c.1245+1G>A) were reclassified as likely benign (LB)/VUS because rescue transcripts were identified in the corresponding genes. Another variant (*EHMT1*:exon2-10 del) was reclassified as a VUS, as exons 2-10 may represent non-constitutive exons, and the deletion (p.8A–549E del) may not affect protein function. Therefore, this variant should not be assigned a PVS1 code.

**Conclusion:**

The presented case studies provide actionable recommendations for identifying rescue transcripts and enhance the understanding of the correct application of PVS1 codes.

## 1 Introduction

High-throughput sequencing, also known as next-generation sequencing (NGS), offers high throughput, efficiency, accuracy, and reduced cost (1). NGS-based variant detection has become an essential component of rare disease diagnostics, including panel sequencing, exome sequencing (ES), and genome sequencing (GS).

As NGS has become more widely used in the diagnosis of rare diseases, an increasing number of variants of uncertain significance (VUS) have been detected, and their interpretation remains challenging (2–4). On this basis, in 2015, the American College of Medical Genetics and Genomics (ACMG) and the Association for Molecular Pathology (AMP) published joint consensus recommendations providing a framework for interpreting sequence variants (5). These guidelines defined 28 criteria for evaluating different forms of variant evidence, each assigned a specific code. Among these, the code PVS1, which describes loss-of-function (LOF) variants, is highly weighted in the evaluation system; therefore, any inappropriate application may lead to misclassification. Consequently, detailed guidance on the specific conditions for its use is essential. To address these challenges, the Clinical Genome (ClinGen) Sequence Variant Interpretation (SVI) Working Group issued additional detailed recommendations on the interpretation of the PVS1 code (6–8). The supplemental guidelines addressed general considerations related to the use of PVS1, including disease mechanisms, the effects of splicing variants, nonsense-mediated decay (NMD), and alternative splicing transcripts. In particular, to promote consistency and clinical relevance regarding different alternatively spliced transcripts, the National Center for Biotechnology Information (NCBI) and the European Molecular Biology Laboratory–European Bioinformatics Institute (EMBL–EBI) collaborated to release reference transcripts through the Matched Annotation from NCBI and EMBL–EBI (MANE) project, as well as MANE Plus Clinical (9). Although MANE Select and MANE Plus Clinical address most clinical concerns, they do not necessarily capture the full biological complexity of disease causation. An illustrative example occurs when a LOF variant in a non-conserved exon of the MANE transcript is detected by ES, yet the individual carrying this variant exhibits no clinical manifestations. In this context, the ACMG Splicing Subgroup introduced the term “rescue transcripts” to guide the interpretation of LOF variants located within non-conserved exons (8). Although LOF variants may cause degradation of the MANE transcript, the functional disruption can be compensated for by the protein produced from rescue transcripts. Based on this concept, certain LOF variants should not be assigned a PVS1 code.

In this study, the properties of four different variants were characterized using *in silico* predictions, protein structural analysis, population frequency data, and clinical observations. Based on these results, three variants (*AMMECR1*:c.220C>T; *GLI2*:c.162_163delCT; *COL11A1*:c.1245+1G>A) were reclassified as likely benign (LB)/VUS because rescue transcripts were identified in the corresponding genes. Another variant (*EHMT1*:exon2-10 del) was reclassified as a VUS, as exons 2-10 may represent non-constitutive exons, and the deletion (p.8A−549E del) may not affect protein function. Therefore, this variant should not be assigned a PVS1 code.

## 2 Materials and methods

### 2.1 Cytomegalovirus (CMV) DNA quantitative assay

The CMV DNA quantitative assay was performed using a real-time PCR system (CFX96, Bio-Rad, Hercules, CA, USA) designed to amplify and qualitatively detect CMV DNA directly from amniotic cells and amniotic fluid without the need for separate nucleic acid extraction (Z-0D-0022-02-A, Liferiver, Shanghai, China).

### 2.2 Analysis of karyotyping in amniotic fluid cells

Ultrasound-guided amniocentesis was performed to collect amniotic fluid cells. Following cultivation, harvesting, slide preparation, and banding, karyotypes displaying 350–550 bands in the metaphase stage were obtained and analyzed using an automated Zeiss microscope (Imager.Z2, Zeiss, Oberkochen, Germany).

### 2.3 Chromosomal microarray analysis (CMA) assay

The CMA procedure was conducted as previously described. Briefly, genomic DNA (gDNA) isolated from blood was digested with restriction enzymes, labeled, purified, and hybridized for 24–40 h at 67 °C. Microarray washing, scanning, feature extraction, and data analysis were performed using Agilent CytoGenomics or Agilent Genomic Workbench software (Agilent Technologies, Santa Clara, CA, USA). Reference gDNA underwent the same experimental procedure.

### 2.4 Exome sequencing (ES)

Total DNA was extracted from blood using a Tiangen kit (DP348, Tiangen, Beijing, China). A total of 1.0 μg of genomic DNA was used as input for library preparation. Sequencing libraries were prepared using the Agilent SureSelect Human All Exon Kit v6 (Agilent Technologies, CA, USA) and sequenced with 2 × 150 bp paired-end reads on the Illumina NovaSeq 6000 system (Illumina, San Diego, CA, USA). Each sample generated more than 10 GB of raw data, with over 90% of bases achieving a Phred quality score > 30. The mean coverage of the genome was × 100, and the minimum coverage of × 10 reached approximately 99%.

The raw reads were aligned to the GRCh37 reference genome using the Burrows–Wheeler Aligner software. Single nucleotide polymorphisms (SNPs) and short insertions/deletions (indels) were identified using SAMtools and GATK software. Variants were annotated using the online analysis platform FLASH ANALYSIS (Geneyx Analysis software_Version 5.15, Shanghai, China). Phenotype-driven analysis of the submitted specimens was conducted, and candidate pathogenic variants were identified by referring to the OMIM, HGMD, ClinVar, MITOMAP, and PubMed databases and relevant literature. Variant interpretation was performed in accordance with the guidelines of the American College of Medical Genetics and Genomics and the Association for Molecular Pathology (ACMG/AMP) for the classification of sequence variants.

### 2.5 Protein 3D structure modeling

Three-dimensional (3D) protein models corresponding to the MANE transcripts of *AMMECR1, COL11A1, EHMT1*, and *GLI2* were obtained from the AlphaFold Protein Database. The altered protein structures for these same genes were modeled using AlphaFold3 (10). The distribution of pathogenic or likely pathogenic (P/LP) missense variants in the genome was examined using the AlphaMissense heat map (11, 12). Visualization of the models was performed with PyMOL 3.1 (The PyMOL Molecular Graphics System, Version 3.0, Schrödinger, LLC), which was used to align wild-type and mutant 3D models.

## 3 Results

### 3.1 Null variants located within non-conserved CDS/ exons in MANE transcripts may not be assigned a PVS1 code

Owing to the limited clinical information and the inability to evaluate organ function in the fetus, the functional impact of many detected variants could not be confirmed phenotypically (13, 14). More generally, many of these variants were *de novo*, and relevant experimental data were unavailable. Therefore, a comprehensive assessment of unknown variants could only be achieved by applying multiple predictive criteria based on existing knowledge of related variants. For nonsense or frameshift variants located within non-conserved coding sequence (CDS)/exons, it is essential to determine whether a rescue transcript exists within the gene (8), as the gene's function may still be maintained by rescue transcripts encoding a full-length protein that retained key domains.

**Figure 1:**
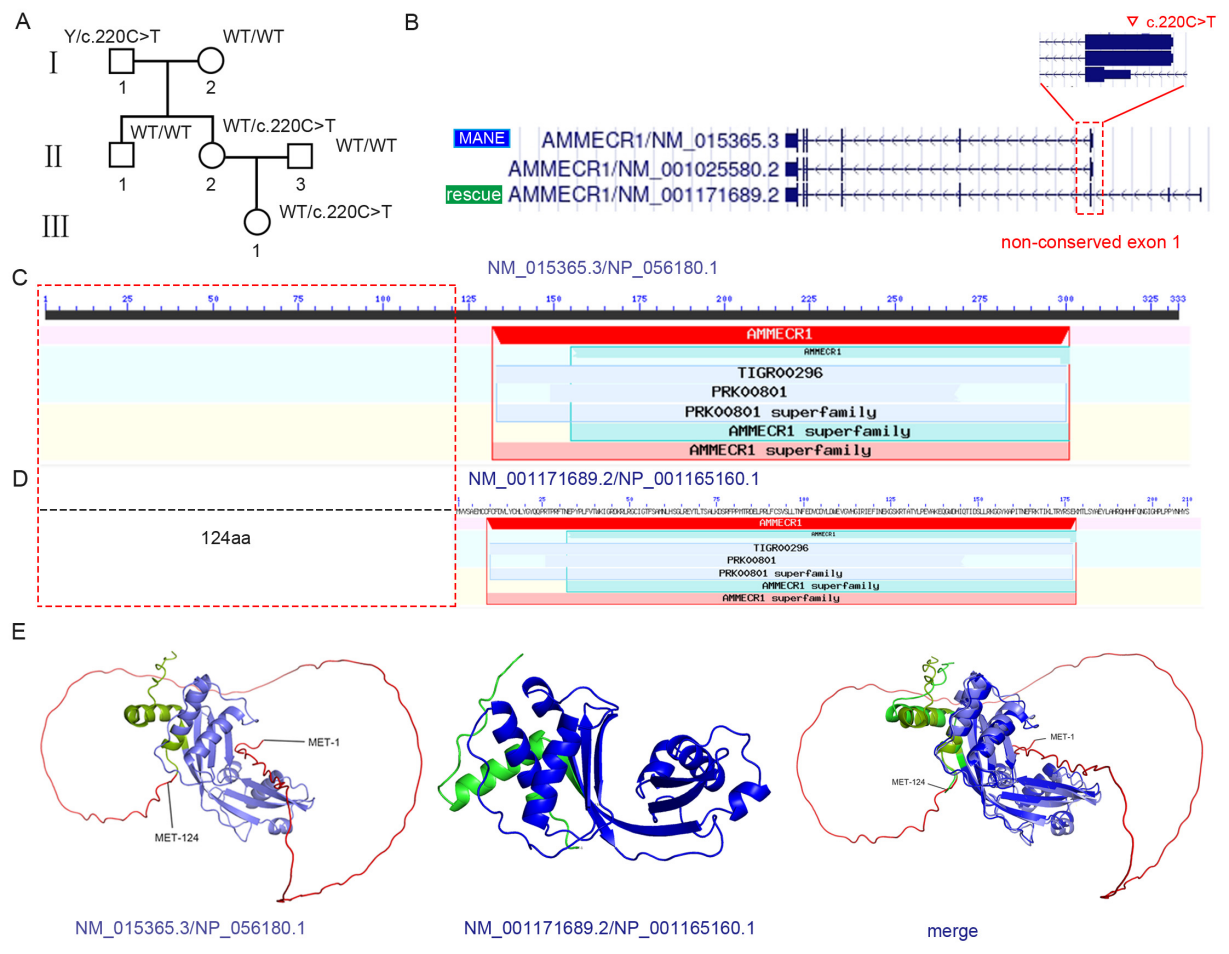
The absence of a non-conserved CDS within a single exon may not disrupt gene function. **(A)** Pedigree of the family. The *AMMECR1* c.220C>T (p.Q74*) variant was identified in the fetus, mother, and grandfather, while other family members were WT. **(B)** Comparison between the MANE transcript (NM_015365.3) and the rescue transcript (NM_001171689.2) showing a non-conserved CDS region. Red dashed box: non-conserved CDS sequence. MANE: Matched Annotation from NCBI and EMBL–EBI. Inverted triangle: variant site. “Rescue”: rescue transcript. **(C)** Two-dimensional domain diagram of the NP_056180.1 protein derived from the NCBI Conserved Domain Database. **(D)** Two-dimensional domain diagram of the NP_001165160.1 protein derived from the NCBI Conserved Domain Database. Red dashed box: non-conserved region (1-124 aa) located at the N-terminus. **(E)** Three-dimensional structures of NP_056180.1 and NP_001165160.1 proteins generated using AlphaFold3. Red sequence: non-conserved region (1-124 aa). Light blue sequence: AMMECR1 superfamily. Light green sequence: other amino acids **(left)**. Blue sequence: AMMECR1 superfamily. Green sequence: other amino acids **(middle)**. Structural alignment between NP_056180.1 and NP_001165160.1 **(right)**.

Case 1: The first pregnant woman, at 23 weeks and 6 days of gestation, was found by B-ultrasound to be carrying a fetus with a suspected cleft palate. Therefore, amniotic fluid cells were collected to detect possible genetic defects. Tests for CMV DNA, cytogenetic karyotyping (46,XX), and CMA all yielded negative results. In addition, a trio-based exome sequencing (Trio-ES) test was performed, which revealed a potential variant: *AMMECR1* (HGNC:467), chrX:109561080 (GRCh37), exon 1, NM_015365.3 (MANE transcript): c.220C>T, p.Q74^*^. This variant was initially classified as likely pathogenic (PVS1 + PM2_Supporting) by the AI-based analysis software. The *AMMECR1* gene was associated with midface hypoplasia, hearing impairment, oval ptosis, and renal calcification (OMIM: 300990, X-linked recessive [XLR], near-complete penetrance). The variant was inherited from the mother (46,XX), who exhibited no clinical manifestations (Figure 1A). In addition, Sanger sequencing revealed that the uncle and grandmother were wild type, while the grandfather was hemizygous for the variant but displayed no disease-related phenotypes (Supplementary Figure 1A). The gnomAD-exome allele frequency was 0.0000084, with one heterozygous female identified in the European (non-Finnish) population. Furthermore, LOF was recognized as a known pathogenic mechanism of the *AMMECR1* gene (15–17). In addition to the MANE transcript (NM_015365.3), the *AMMECR1* gene had two alternatively spliced transcripts listed in the UCSC database. The variant *AMMECR1*: c.220C>T occurred in exon 1 of NM_015365.3; however, exon 1 was a non-conserved exon (Supplementary Figure 2), and P/LP LOF variants were absent in the non-conserved CDS. A search of the gnomAD database revealed that 1 female carriers of truncating variant had been recorded in non-conserved CDS. Moreover, the non-conserved CDS was not present in the candidate rescue transcript (NM_001171689.2) (Figure 1B). Next, The MANE transcript and the candidate rescue transcript were both expressed in brain tissue. According to the GTEx database (Supplementary Figure 3) (18). Furthermore, data from the NCBI Conserved Domain Database (19), AlphaFold3, and the AlphaMissense Database showed that, within the protein (NP_056180.1) corresponding to the MANE transcript (NM_015365.3), amino acids 1–124 (derived from the non-conserved CDS) were not located within any critical functional domains (Figures 1C, D; Supplementary Figures 1B–D). The deletion of residues 1–124 had minimal impact on the three-dimensional structure of the protein (Figure 1E). Based on these findings, NM_001171689.2 was identified as a rescue transcript, and the PVS1 code should not be applied to this variant. Finally, this variant was classified as LB: BS4 (−4), PM2_Supporting (+1) (20). The mother elected to continue the pregnancy and gave birth to a healthy daughter.

**Figure 2:**
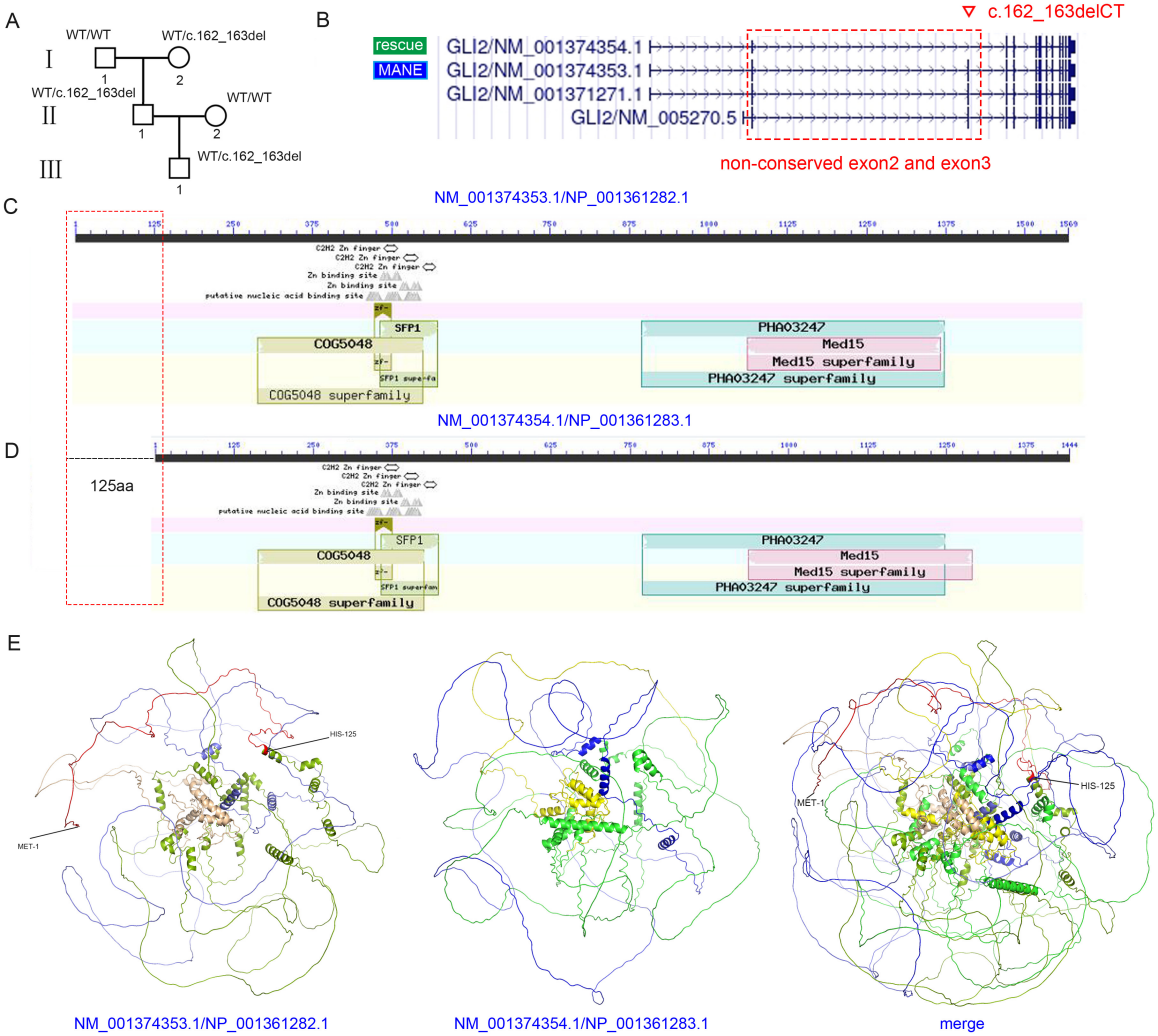
The absence of several non-conserved exons may not disrupt gene function. **(A)** Pedigree of the family. The *GLI2* c.162_163delCT variant was identified in the fetus, father, and grandmother, while other family members were WT. **(B)** Comparison between the MANE transcript (NM_001374353.1) and the rescue transcript (NM_001374354.1), showing the absence of exons 2 and 3 in the rescue transcript. Red dashed box: non-conserved exons 2 and 3. MANE: Matched Annotation from NCBI and EMBL–EBI. Inverted triangle: variant site. “Rescue”: rescue transcript. **(C)** Two-dimensional domain diagram of the NP_001361282.1 protein derived from the NCBI Conserved Domain Database. **(D)** Two-dimensional domain diagram of the NP_001361283.1 protein derived from the NCBI Conserved Domain Database. Red dashed box: non-conserved region (1-125 aa) located at the N-terminus. **(E)** Three-dimensional structures of NP_001361282.1 and NP_001361283.1 proteins generated using AlphaFold3. Red sequence: non-conserved amino acid region (1-125 aa). Light yellow sequence: COG5048 superfamily. Light blue sequence: PHA03247 superfamily. Light green sequence: other amino acids **(left)**. Yellow sequence: COG5048 superfamily. Blue sequence: PHA03247 superfamily. Green sequence: other amino acids **(middle)**. Structural alignment between NP_001361282.1 and NP_001361283.1 **(right)**.

Case 2: The second pregnant woman, at 23 weeks of gestation, was found during routine prenatal examination to be carrying a fetus with a single umbilical artery and a fused kidney. Therefore, amniotic fluid cells were collected to detect possible genetic defects. Tests for CMV DNA, cytogenetic karyotyping, and CMA all yielded negative results. In addition, a Trio-ES test was performed, revealed a potential variant: *GLI2* (HGNC:4318), chr2:121684950_121684951 (GRCh37), exon 3, NM_001374353.1 (MANE transcript), c.162_163delCT: p.L55Afs^*^10, which was initially classified as likely pathogenic (PVS1 + PM2_Supporting) by the AI-based analysis software. The *GLI2* gene was associated with Culler-Jones syndrome (OMIM: 615849, autosomal dominant [AD], incomplete penetrance and variable expressivity) and Holoprosencephaly 9 (OMIM: 610829, AD, incomplete penetrance and variable expressivity). The variant was inherited from the father, who exhibited no disease-associated phenotypes (Figure 2A). In addition, Sanger sequencing revealed that the grandmother was heterozygous and the grandfather was wild type for the variant, yet the grandmother displayed no disease-related phenotypes (Supplementary Figure 4A). The gnomAD-exome allele frequency was 0.000012, and the c.162_163delCT variant was observed in three East Asian individuals (2 Female, 1 Male). Furthermore, according to the PubMed database, LOF variants in *GLI2* were known to be pathogenic (21, 22). In addition to the MANE transcript (NM_001374353.1), three alternatively spliced transcripts were identified in the UCSC database (Figure 2B). The variant *GLI2*: c.162_163delCT occurred in exon 3; however, exons 2 and 3 were non-conserved exons (Supplementary Figure 5), and P/LP LOF variants were not present in these regions. Importantly, a search of the gnomAD database revealed that 13 male carriers and 3 female carriers of truncating variants had been recorded in non-conserved exon2 and exon3. Moreover, the non-conserved exons 2 and 3 were absent in the candidate rescue transcript (NM_001374354.1). Although the variant was listed in ClinVar (Variation ID: 817375) and annotated as likely pathogenic, it had not been reported in any published cases involving *GLI2*-related disorders. Additionally, the same expression levels were observed between the MANE transcript and the candidate rescue transcript across multiple tissues according to the GTEx database (Supplementary Figure 8). Finally, analysis using the NCBI Conserved Domain Database, AlphaFold3, and the AlphaMissense Database indicated that, within the protein (NP_001361282.1) corresponding to the MANE transcript (NM_001374353.1), amino acids 1–125 did not form part of any critical functional domains (Figures 2C, D; Supplementary Figures 4B–D). The removal of residues 1–125 had minimal impact on the protein's three-dimensional structure (Figure 2E). Based on these findings, NM_001374354.1 was identified as a rescue transcript, and the PVS1 code should not be applied to this variant. Finally, this variant was classified as VUS: PM2_Supporting (+1). The mother elected to continue her pregnancy and gave birth to a healthy son.

### 3.2 Splicing variants located within non-conserved exons in MANE transcripts may not be assigned a PVS1 code

According to the recommendations of the SVI Splicing Subgroup, for splice donor or acceptor ±1/2 dinucleotide variants, it was essential to determine whether the predominant splicing alteration was predicted to be in-frame or out-of-frame using a bioinformatic prediction tool or an RNA reverse transcription assay. However, when splicing variants occurred within non-conserved exons in MANE transcripts, even if the predicted alteration was out-of-frame, such variants should not be assigned a PVS1 code.

**Figure 3:**
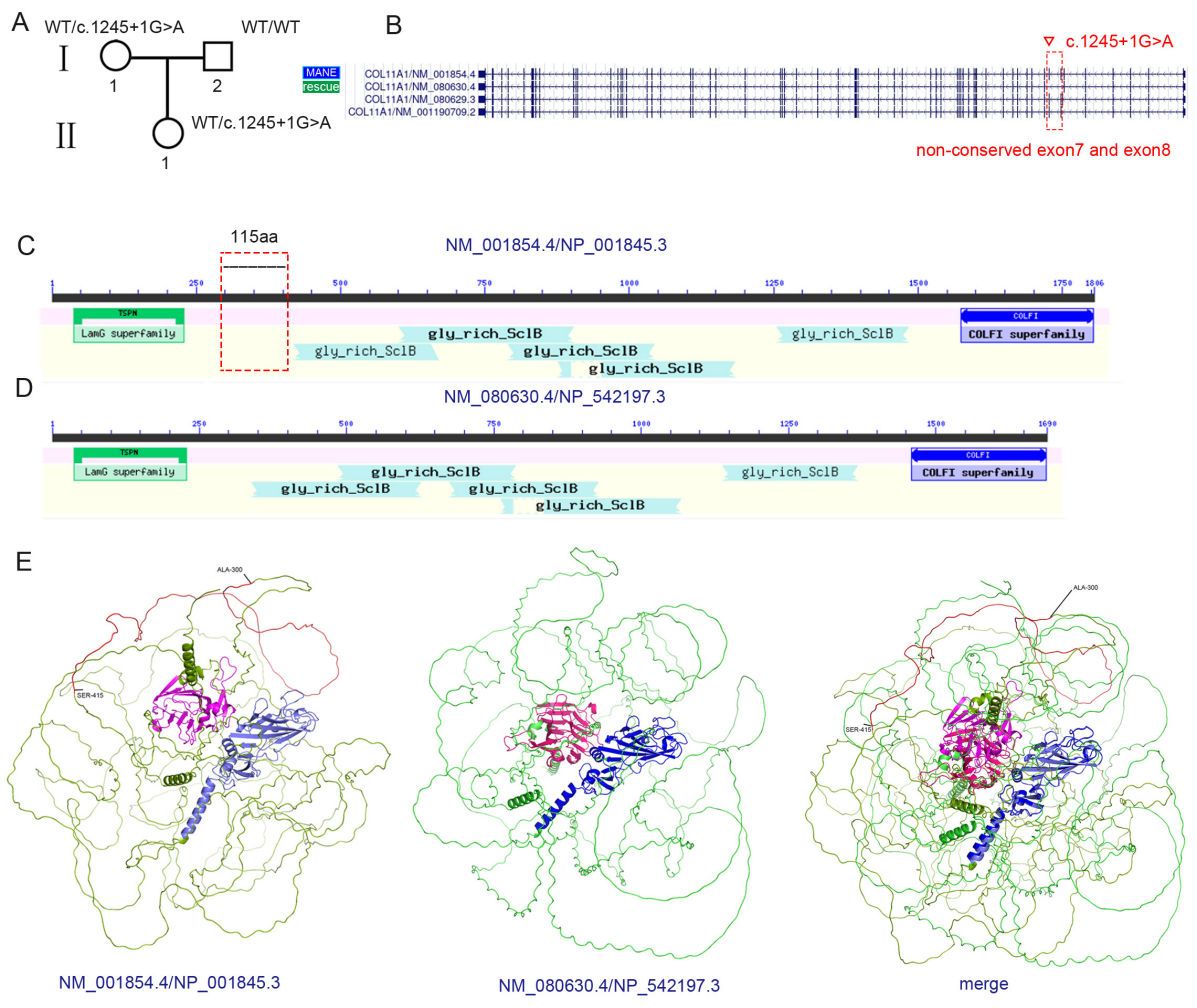
Splicing variants in non-conserved exons may not disrupt gene function. **(A)** Pedigree of the family. The *COL11A1* c.1245+1G>A variant was identified in the mother and daughter, while the father was WT. **(B)** Comparison between the MANE transcript (NM_001854.4) and the rescue transcript (NM_080630.4), showing that the rescue transcript lacks exons 7 and 8. Red dashed box: non-conserved exons 7 and 8. MANE: Matched Annotation from NCBI and EMBL–EBI. Inverted triangle: variant site. “Rescue”: rescue transcript. **(C)** Two-dimensional domain diagram of the NP_001845.3 protein derived from the NCBI Conserved Domain Database. Red dashed box: non-conserved region located in the middle of the protein. **(D)** Two-dimensional domain diagram of the NP_542197.3 protein derived from the NCBI Conserved Domain Database. **(E)** Three-dimensional structures of NP_001845.3 and NP_542197.3 proteins generated using AlphaFold3. Red sequence: non-conserved amino acid region (300-415 aa). Light blue sequence: COLFI superfamily. Light pink sequence: TSPN domains. Light green sequence: other amino acids **(left)**. Blue sequence: COLFI superfamily. Pink sequence: TSPN domains. Green sequence: other amino acids **(middle)**. Structural alignment between NP_001845.3 and NP_542197.3 **(right)**.

Case 3: The third pregnant woman, at 25 weeks and 3 days of gestation, was found to be carrying a fetus with intrauterine growth restriction and focal strong echogenicity in the left ventricle (left ventricular sinus and aortic vestibule). Therefore, amniotic fluid cells were collected to detect possible genetic defects. Tests for CMV DNA, cytogenetic karyotyping, and CMA all yielded negative results. In addition, a Trio-ES test was performed, revealed a potential variant: *COL11A1* (HGNC:2186), chr1:103488297 (GRCh37), intron 8, NM_001854.4 (MANE transcript): c.1245+1G>A, p.?, which was initially classified as likely pathogenic (PVS1 + PM2_Supporting) by the AI-based analysis software. *COL11A1* was associated with deafness, type 37 (OMIM: 618533, AD, high penetrance and variable expressivity); fibrochondrogenesis 1 (OMIM: 228520, autosomal recessive [AR]); Marshall syndrome (OMIM: 154780, AD, high penetrance and variable expressivity); and Stickler syndrome, type II (OMIM: 604841, AD, high penetrance and variable expressivity). The variant was inherited from the mother, who exhibited no disease-associated phenotypes (Figure 3A; Supplementary Figure 7A). The gnomAD-exome allele frequency was 0.000008083, with two heterozygous carriers identified in the East Asian populations (1 Female,1 Male), respectively; the internal database included 11 heterozygous carriers without any disease-related characteristics. Furthermore, the SpliceAI prediction results indicated that this variant led to the loss of the splicing donor site (score: 1.0) (Supplementary Figure 7B) in the MANE transcript (NM_001854.4) and the autoPVS1 prediction results indicated that this variant triggered NMD (Supplementary Figure 7C) (23, 24). Analysis using the third tool RNA Splicer (https://rddc.tsinghua-gd.org/zh/tool/rna-splicer) revealed two distinct splicing consequences: 1. Intron retention: The DanQ score rose from 0.6897 to 0.952, indicating a high probability of intron retention. A 28-base pair segment would insert into the mRNA and lead to a frameshift. 2. Exon 8 skipping: This variant would produce an in-frame deletion, which resulted in a truncated protein product (Supplementary Figure 8). Additionally, LOF variants in *COL11A1* were known to be pathogenic (25–28). The variant had also been submitted to the ClinVar database (Variation ID: 1324096) and classified as LP. However, the reported classifications in the literature were inconsistent, as the variant did not show co-segregation with disease phenotypes (29–32). In addition to the MANE transcript (NM_001854.4), three alternatively spliced transcripts were identified in the UCSC database. The variant *COL11A1*:c.1245+1G>A occurred in intron 8, but exons 7 and 8 were alternatively splicing exons (Figure 3B; Supplementary Figure 9), and P/LP LOF variants were absent in these regions. Importantly, a search of the gnomAD database revealed that 4 male carriers and 4 female carriers of truncating variants had been recorded in non-conserved exon7 and exon8. Moreover, exons 7 and 8 were absent in the candidate rescue transcript (NM_080630.4). Both the MANE transcript and the candidate rescue transcript were highly expressed in cultured fibroblast cells according to the GTEx database (33, 34) (Supplementary Figure 10). Finally, analysis using the NCBI Conserved Domain Database, AlphaFold3, and the AlphaMissense Database revealed that, within the protein (NP_001845.3) corresponding to the MANE transcript (NM_001854.4), amino acids 300–415 (encoded by exons 7 and 8) did not belong to any critical functional domains (Figures 3C, D; Supplementary Figures 7D–F), and the deletion of residues 300–415 had minimal impact on the protein's three-dimensional structure (Figure 3E). Despite the degradation of the MANE transcript (NM_001854.4) caused by NMD activation due to the c.1245+1G>A variant, the protein encoded by the rescue transcript (NM_080630.4) was able to function normally.

Therefore, when fully referencing the prediction results of three tools, there may be two allocation schemes for this variant: 1. despite the degradation of the MANE transcript (NM_001854.4) caused by NMD activation due to the c.1245+1G>A variant, the protein encoded by the rescue transcript (NM_080630.4) was able to function normally. Therefore, the c.1245+1G>A variant should not be assigned a PVS1 code.2.when the c.1245+1G>A variant would result in the skipping of exon 8, producing an in-frame deletion that would lead to a truncated protein (331-415aa del). In this case, a PVS1_moderate code (+2) should be assigned to the variant. Finally, the variant was classified as VUS. The mother elected to continue her pregnancy and gave birth to a healthy daughter.

### 3.3 The deletion of multiple exons encoding non-critical domains may not necessarily affect gene function

For more than a decade, CMA had been widely used in clinical settings to detect genomic imbalances with greater resolution than conventional cytogenetic methods, such as G-banded karyotyping, in prenatal diagnosis (35). However, owing to the incomplete genome-wide coverage of CMA probes and the absence of probe coverage in certain genomic regions, missed detections remained common.

**Figure 4:**
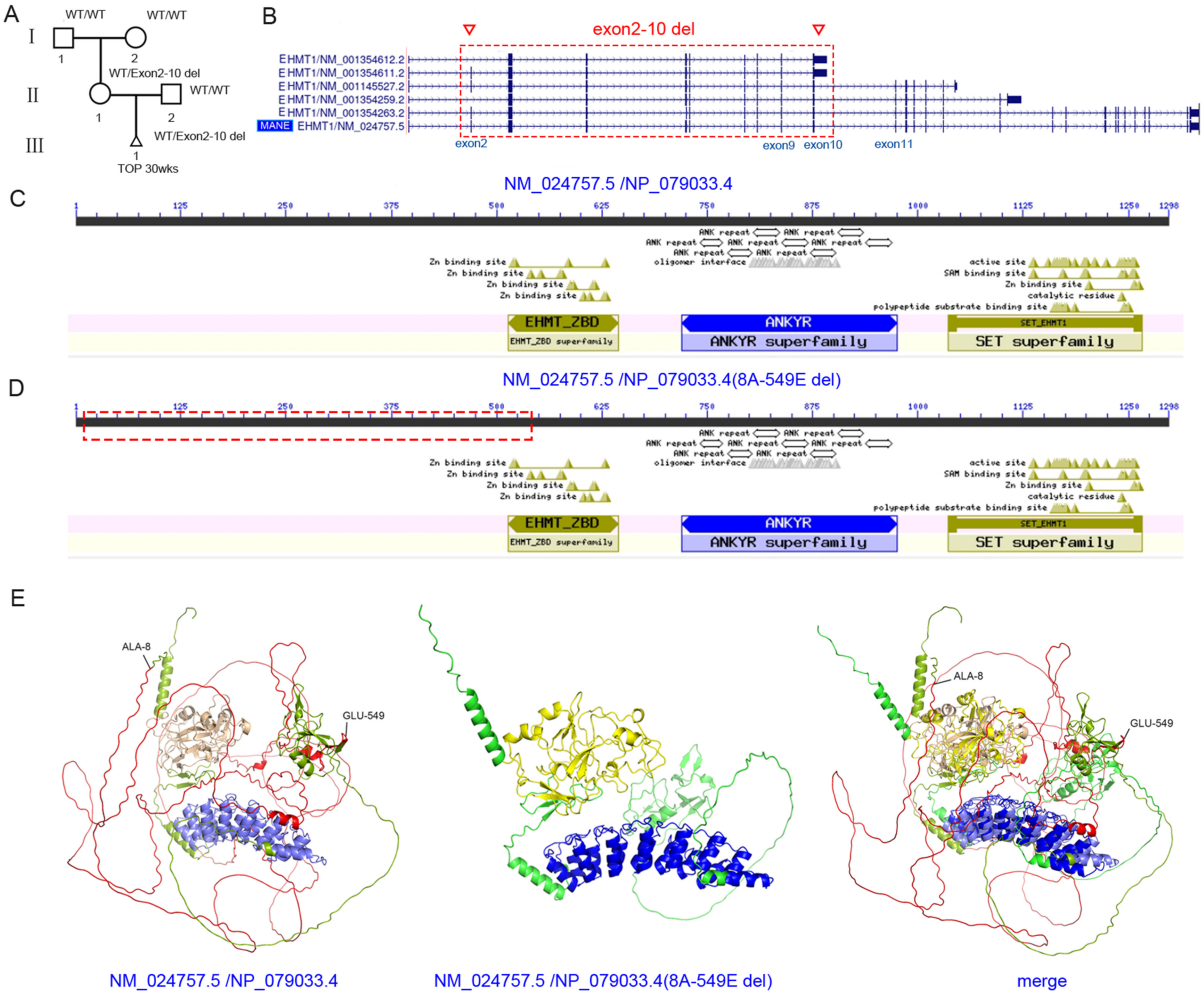
Large in-frame deletions may not disrupt gene function. **(A)** Pedigree of the family. The *EHMT1* (NM_024757.5) exon 2-10 deletion was identified in the fetus and mother, while the father, grandfather, and grandmother were wild type (WT). TOP: termination of pregnancy. wks: weeks. **(B)** Exons 2-10 (NM_024757.5) were not conserved across all transcripts. MANE: Matched Annotation from NCBI and EMBL–EBI. Inverted triangle: variant site. **(C)** A two-dimensional domain diagram of NP_079033.4 protein, derived from NCBI-Conserved Domain Database. **(D)** A two-dimensional domain diagram of NP_079033.4 protein, derived from NCBI-Conserved Domain Database. Red dashed box: the deletion of amino acid sequences (8-549aa) located N-terminal. **(E)** Three-dimensional structures of NP_079033.4 proteins generated using AlphaFold3. Red sequence: deleted amino acid region (8-549 aa). Light blue sequence: ANKYR superfamily. Light yellow sequence: SET superfamily. Light green sequence: other amino acids **(left)**. The three-dimensional structure of NP_079033.4 (with deletion of 8-549 aa) generated using AlphaFold3. Blue sequence: ANKYR superfamily. Yellow sequence: SET superfamily. Green sequence: other amino acids **(middle)**. Structural alignment between NP_079033.4 and NP_079033.4 (with deletion of 8-549 aa) **(right)**.

Case 4: The fourth pregnant woman, aged 34 years and at 19 weeks and 3 days of gestation, underwent prenatal screening using cell-free DNA testing to detect possible genetic defects, which revealed a reduced number of sex chromosomes. To further confirm the number of sex chromosomes, CMA testing was performed on amniotic fluid cells. Tests for CMV DNA and cytogenetic karyotyping yielded negative results. CMA (CGX SNP v1.1, Agilent, G4884A) showed no abnormalities in the number of sex chromosomes; however, another variant was detected: chr9:140584646_140654963 × 1 (GRCh37), *EHMT1* (HGNC:24650), NM_024757.5 (MANE transcript): exon 2-9 deletion (of 27 exons) (Supplementary Figure 11A), which was expected to result in a frameshift and was initially classified as likely pathogenic (PVS1 + PM2_Supporting) by the AI-based analysis software. *EHMT1* (haploinsufficiency score: 3) was associated with Kleefstra syndrome type 1 (OMIM: 610253, AD, incomplete penetrance). The pregnant woman choose to terminate the pregnancy (Figure 4A). Furthermore, CMA testing was performed on the pregnant woman and her husband. The results showed that the pregnant woman carried the variant, although no disease-related phenotypes were observed. To further determine whether the variant was present in other family members, additional analyses were conducted on the pregnant woman and her parents using ES for DNA testing. The results revealed a potential variant: chr9:140591675_140657373 × 1 (GRCh37), *EHMT1*, NM_024757.5, exon 2–10 deletion, p.8A-549E del. The pregnant woman was heterozygous, whereas her parents were wild type (Supplementary Figure 11B). Furthermore, deletion of exon 10 was confirmed by qPCR (Supplementary Figure 12). This variant resulted in an in-frame deletion in the *EHMT1* gene, affecting more than 10% of the protein. In addition, querying the ClinVar database revealed no P/LP missense variants within the 1-549 amino acid region, suggested that this was not a critical region (36). Moreover, *EHMT1* exon 2-10 (NM_024757.5) was not fully conserved across the other five transcripts according to the UCSC database (Figure 4B; Supplementary Figure 8). Finally, analysis using the NCBI Conserved Domain Database, AlphaFold3, and AlphaMissense Database indicated that amino acids 8-549 were not located within any critical protein domain (Figures 4C, D; Supplementary Figures 11C, D), and the removal of residues 8-549 had minimal impact on the protein's three-dimensional structure (Figure 4E). Based on these findings, *EHMT1* (NM_024757.5) exon 2–10 may represent non-constitutive exons, and the p.8A_549Edel variant was unlikely to affect protein activity. Therefore, this variant was classified as a VUS: PM2_Supporting (+1), PM4 (+2).

## 4 Discussion

In this study, four different putative LOF variants were identified in four unrelated families using Trio-ES. These variants were initially classified as likely pathogenic (PVS1 + PM2_Supporting) by the AI-based analysis software. However, following manual review, none of these variants met the criteria for applying the PVS1 code and were consequently downgraded to LB or VUS. Furthermore, co-segregation analysis revealed that individuals carrying these variants exhibited no disease-related phenotypes, confirming the non-pathogenic nature of the variants. Mechanistically, although variant-induced degradation of the MANE transcript occurred through NMD, the proteins encoded by the rescue transcripts retained the essential structural and functional elements of the wild-type protein. Their three-dimensional conformations were preserved, allowing them to maintain normal function (Table 1) (37). Collectively, these cases provided updated clinical evidence supporting the concept of rescue transcripts and may assist clinical geneticists in applying the PVS1 code with greater accuracy and confidence.

**Table 1:** Steps for identifying a candidate rescue transcript.

Description	Available databases
1. There are several annotated alternative splicing transcripts in the gene.	UCSC, NCBI
2. The candidate rescue transcript is physiologically expressed at levels that meet the needs of the organism or has a similar level of expression to the MANE transcript.	UCSC, GTEx
3. The candidate rescue transcript lacks non-conserved CDS/exons, and the non-conserved CDS/exons in the MANE transcript conform to the absence of likely pathogenic/pathogenic stop-gain variants and exon-level deletions, or/and LoF variants in non-conserved CDS/exons are frequent in the general population.	UCSC, HGMD, ClinVar, DECIPHER, ClinGen, gnomAD, and PubMed
4. The protein sequence encoded by the non-conserved CDS/exons in the MANE transcript does not fall within the critical protein domain.	NCBI Conserved Domain Database, AlphaFold3, the AlphaMissense Database, and PubMed
5. The removal of protein sequences encoded by the non-conserved CDS/exons in the MANE transcript does not disrupt the protein 3D structure.	NCBI Conserved Domain Database, AlphaFold3, the AlphaMissense Database, and PubMed

These annotated physiological alternative splicing transcripts were termed ‘rescue transcripts '. These rescue transcripts were physiologically expressed at levels that met the needs of the organism, maintained the reading frame and encoded critical protein domains (38). In the past, it was difficult to determine the minimum level of gene expression required by the human body. Therefore, identifying this level was an important area for future research. Moreover, in rescue transcripts, non-conserved exons were either absent or had been converted into non-coding sequences; in both cases, this resulted in the loss of a portion of the protein sequence that did not encode key domains. In addition, non-conserved exons were expected to lack likely pathogenic/pathogenic stop-gain variants and exon-level deletions in databases related to human diseases. Furthermore, LoF variants in non-conserved CDS/exons were frequent in the general population. The ClinGen SVI Splicing Subgroup suggested employing PVS1_N/A for variants that had a credible rescue transcript (8).

Another variant, *COL11A1*:c.1245+2T>C, although listed in the ClinVar database (Variation ID: 1324096), remained controversial, as it was inconsistent with familial co-segregation findings in both previous reports and the present study. Therefore, when referencing P/LP variants from ClinVar, it was advisable to re-evaluate these variants, particularly when the observed genotypes and phenotypes were incongruent.

For more than a decade, copy number variant (CNV) analysis using CMA had been widely applied in clinical practice to detect genomic imbalances with higher resolution than conventional cytogenetic techniques, such as G-banded karyotyping (39–41). In certain cases, exon-focused array designs had also been employed for CNV detection (42). However, because CMA probes did not provide complete genome-wide coverage and lacked probe representation in some genomic regions, missed detections remained common (43, 44). In this study, within family 4, *EHMT1* exon 2-9 deletion (NM_024757.5, 27 exons) was detected using CMA. This variant was predicted to cause a frameshift and was initially classified as LP. By contrast, *EHMT1* exon 2-10 deletion (NM_024757.5, 27 exons) was identified using ES, which revealed an in-frame deletion classified as a VUS. The discrepancy arose because introns 9 and 10 of the *EHMT1* gene lacked probe coverage in the CMA platform, preventing detection of the exon 10 deletion and resulting in an inaccurate variant classification. Therefore, when variants identified through CMA were categorized as VUS, validation through family co-segregation analysis was recommended. Alternatively, NGS techniques such as ES or GS can be used for co-segregation analysis.

## Data Availability

The original contributions presented in the study are included in the article/Supplementary material, further inquiries can be directed to the corresponding author.

## Supplementary material

Supplementary Figure S1 Additional information related to the *AMMECR1* gene. **(A)** Sanger sequencing verification of the variant. Red arrow: variant site. **(B)** Domain distribution in the NP_056180.1 protein. **(C)** Domain distribution in the NP_001165160.1 protein. **(D)** AlphaMissense pathogenicity heat map of the NP_056180.1 protein. Black dashed box: distribution of likely benign/VUS missense variants (11-123 aa).

Supplementary Figure S2 A multi-species comparison of the non-conserved CDS of the *AMMECR1* gene.

Supplementary Figure S3 Expression levels of the MANE transcript (NM_015365.3) and the rescue transcript (NM_001171689.2). **(A)** Expression level of the MANE transcript (NM_015365.3) across different tissues. **(B)** Expression level of the rescue transcript (NM_001171689.2) across different tissues.

Supplementary Figure S4 Additional information related to the *GLI2* gene. **(A)** Sanger sequencing verification of the variant. **(B)** Domain distribution in the NP_001361282.1 protein. **(C)** Domain distribution in the NP_001361283.1 protein. **(D)** AlphaMissense pathogenicity heat map of the NP_001361282.1 protein. Black dashed box: distribution of likely benign/VUS missense variants (2-130 aa).

Supplementary Figure S5 A multi-species comparison of the non-conserved exons of the *GLI2* gene.

Supplementary Figure S6 Expression levels of the MANE transcript (NM_001374353.1) and the rescue transcript (NM_001374354.1). The MANE transcript (NM_001374353.1) and the rescue transcript (NM_001374354.1) showed comparable expression levels.

Supplementary Figure S7 Additional information related to the *COL11A1* gene. **(A)** BAM file visualization of the variant. **(B)** SpliceAI prediction result for the variant. Black box: SpliceAI score = 0.29 (donor gain); SpliceAI score = 1.0 (donor loss). **(C)** AutoPVS1 prediction result for the variant. Exon skipping or use of a cryptic splice site disrupts the reading frame and is predicted to undergo NMD. ACMG classification: PVS1. **(D)** Domain distribution in the NP_001845.3 protein. **(E)** Domain distribution in the NP_542197.3 protein. **(F)** AlphaMissense pathogenicity heat map of the NP_001845.3 protein. Black dashed box: distribution of likely benign/VUS missense variants (243-423 aa).

Supplementary Figure S8 Analysis using the RNA Splicer revealed two distinct splicing consequences: intron retention and exon 8 skipping.

Supplementary Figure S9 a multi-species comparison of the non-conserved exons of the *COL11A1* gene.

Supplementary Figure S10 Expression levels of the MANE transcript (NM_001854.4) and the rescue transcript (NM_080630.4). **(A, B)** Both the MANE transcript (NM_001854.4) and the rescue transcript (NM_080630.4) exhibited high expression specifically in cell-transformed fibroblasts.

Supplementary Figure S11 Additional information related to the *EHMT1* gene. **(A)** Probe distribution in CGX SNP v1.1 (Agilent, G4884A). Solid circles represent detection signals. The purple vertical line indicates preset probe positions, and the solid box indicates regions without probe coverage. **(B)** Reads alignment results for exons 10 and 11 of the *EHMT1* gene. Red dashed box: heterozygous deletion of exon 10 in the mother. **(C)** Domain distribution in the NP_079033.4 protein. **(D)** AlphaMissense pathogenicity heat map of the NP_079033.4 protein. Black dashed box: distribution of likely benign/VUS missense variants (1-510 aa).

Supplementary Figure S12 qPCR verification of the deletion of exon 10 in the *EHMT1* gene.

Supplementary Figure S13 A multi-species comparison of the non-conserved exons of the *EHMT1* gene.
