# Supplementary figures and images for: Correcting PVS1 overestimation: clinical insights into rescue transcripts and variant reclassification in 4 prenatal cases
Source: Front Med (Lausanne). 2026 May 13;13:1799731. doi: 10.3389/fmed.2026.1799731 (PMC13212255; doi:10.3389/fmed.2026.1799731)

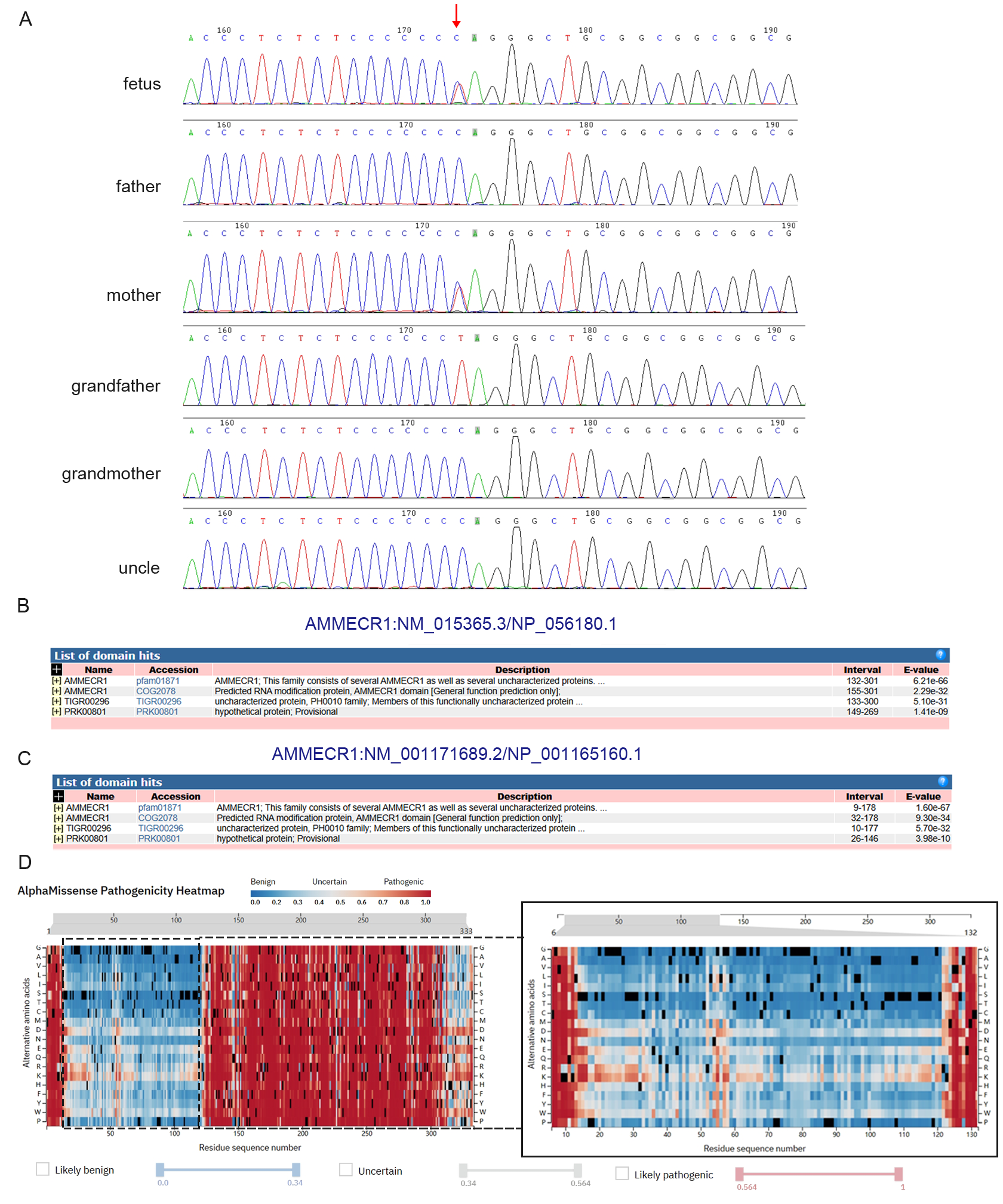

Supplement: Supplementary Figure S1 — Additional information related to the AMMECR1 gene. (A) Sanger sequencing verification of the variant. Red arrow: variant site. (B) Domain distribution in the NP_056180.1 protein. (C) Domain distribution in the NP_001165160.1 protein. (D) AlphaMissense pathogenicity heat map of the NP_056180.1 protein. Black dashed box: distribution of likely benign/VUS missense variants (11-123 aa). [file Image_1.tif]

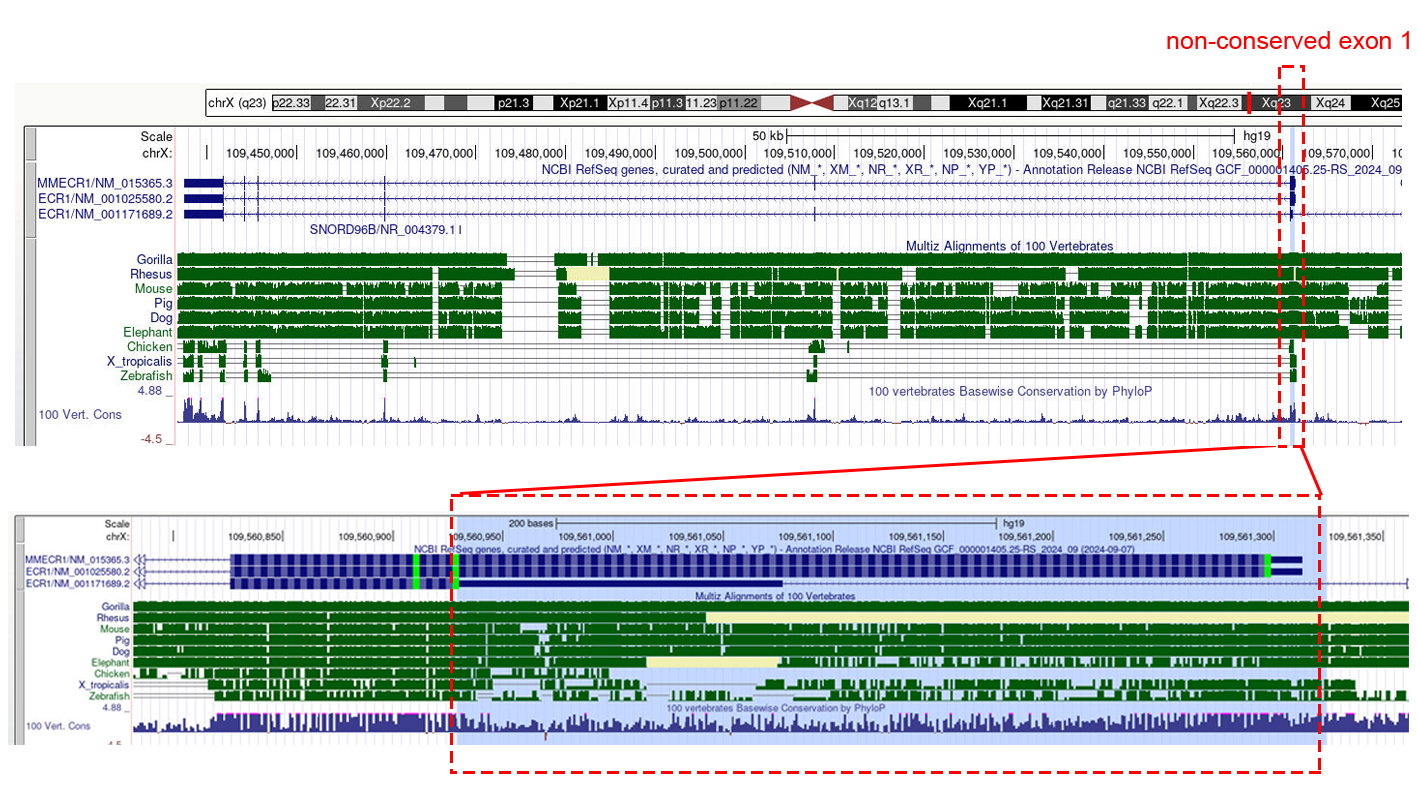

Supplement: Supplementary Figure S2 — A multi-species comparison of the non-conserved CDS of the AMMECR1 gene. [file Image_2.tif]

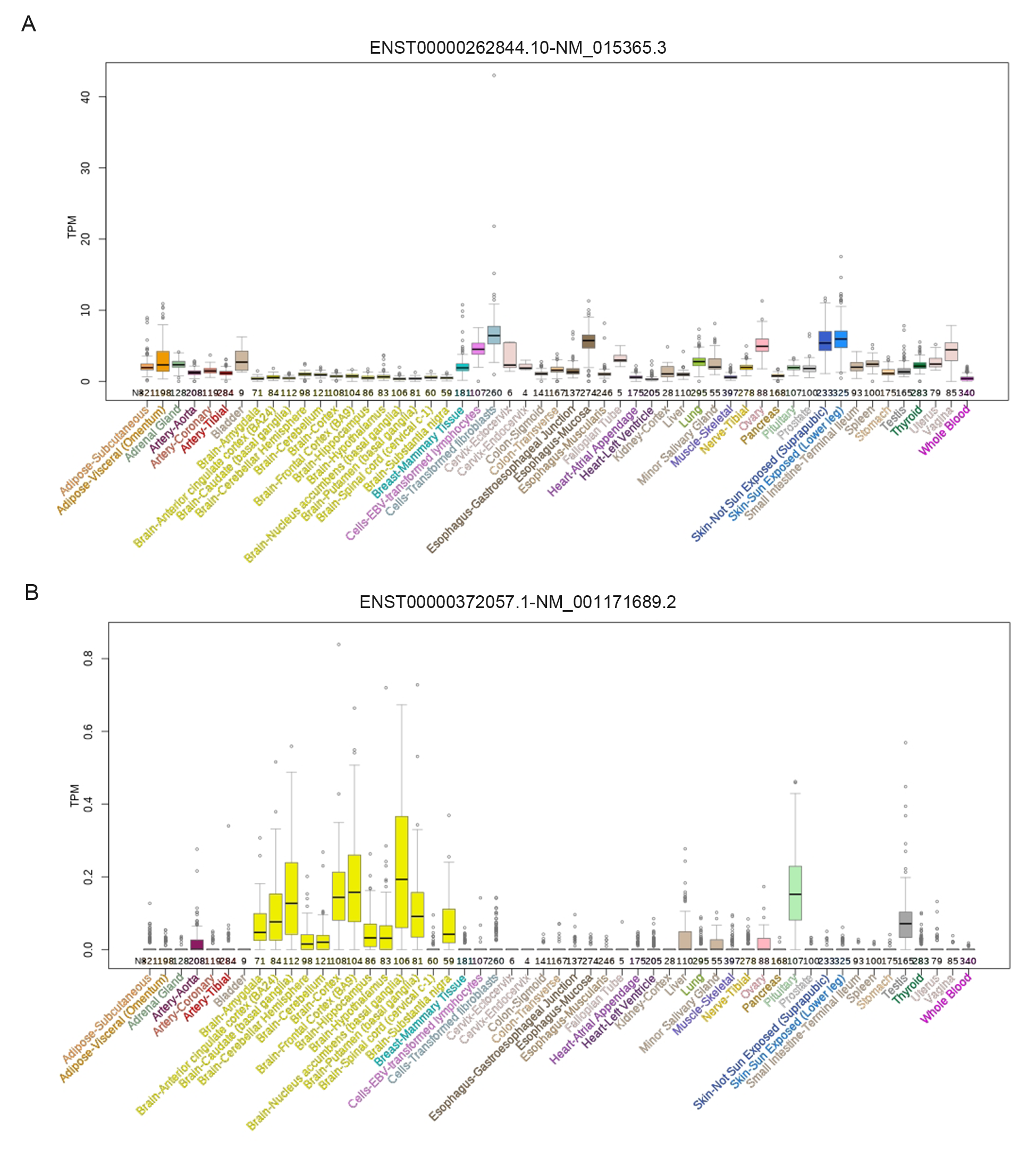

Supplement: Supplementary Figure S3 — Expression levels of the MANE transcript (NM_015365.3) and the rescue transcript (NM_001171689.2). (A) Expression level of the MANE transcript (NM_015365.3) across different tissues. (B) Expression level of the rescue transcript (NM_001171689.2) across different tissues. [file Image_3.tif]

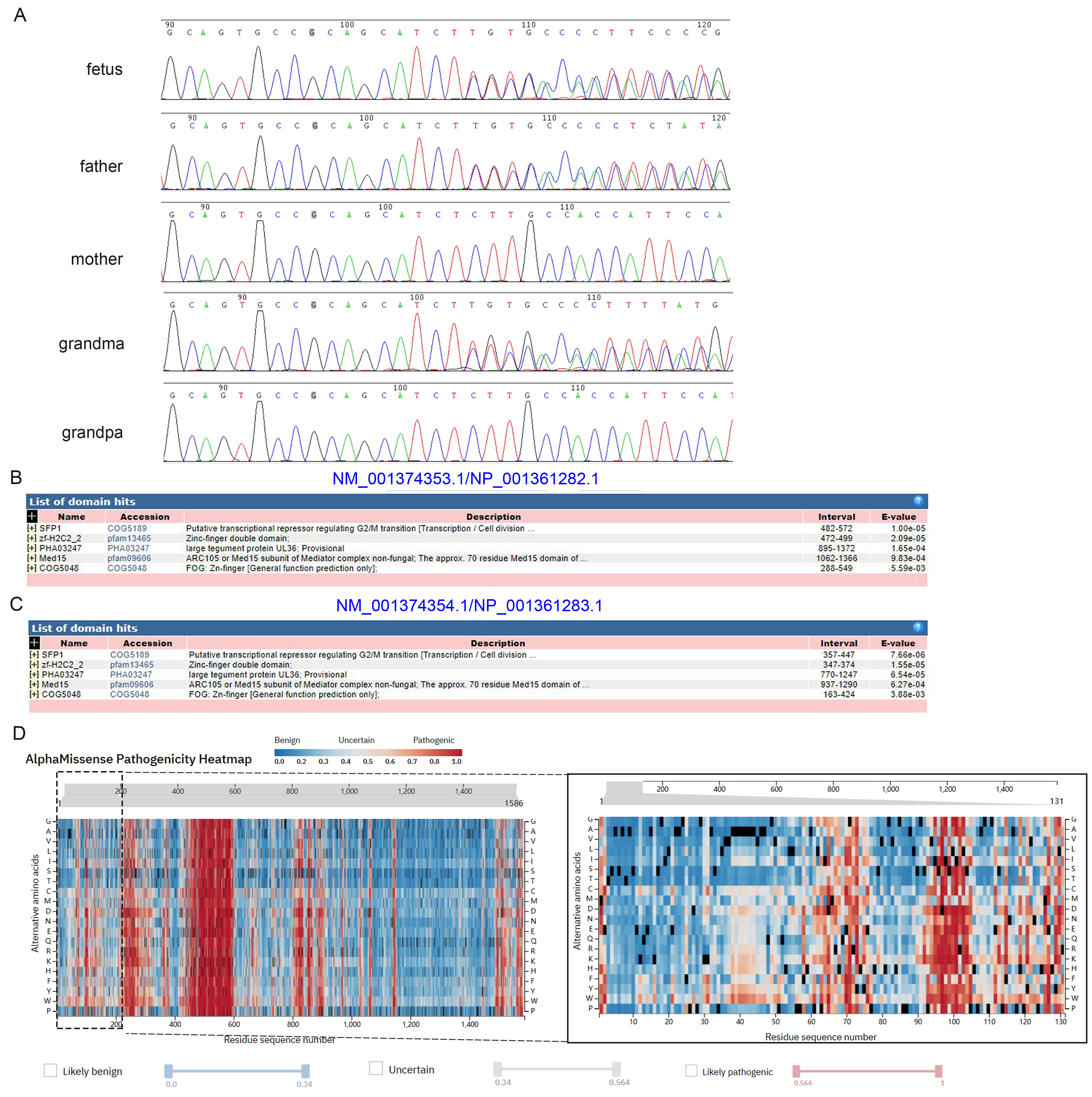

Supplement: Supplementary Figure S4 — Additional information related to the GLI2 gene. (A) Sanger sequencing verification of the variant. (B) Domain distribution in the NP_001361282.1 protein. (C) Domain distribution in the NP_001361283.1 protein. (D) AlphaMissense pathogenicity heat map of the NP_001361282.1 protein. Black dashed box: distribution of likely benign/VUS missense variants (2-130 aa). [file Image_4.tif]

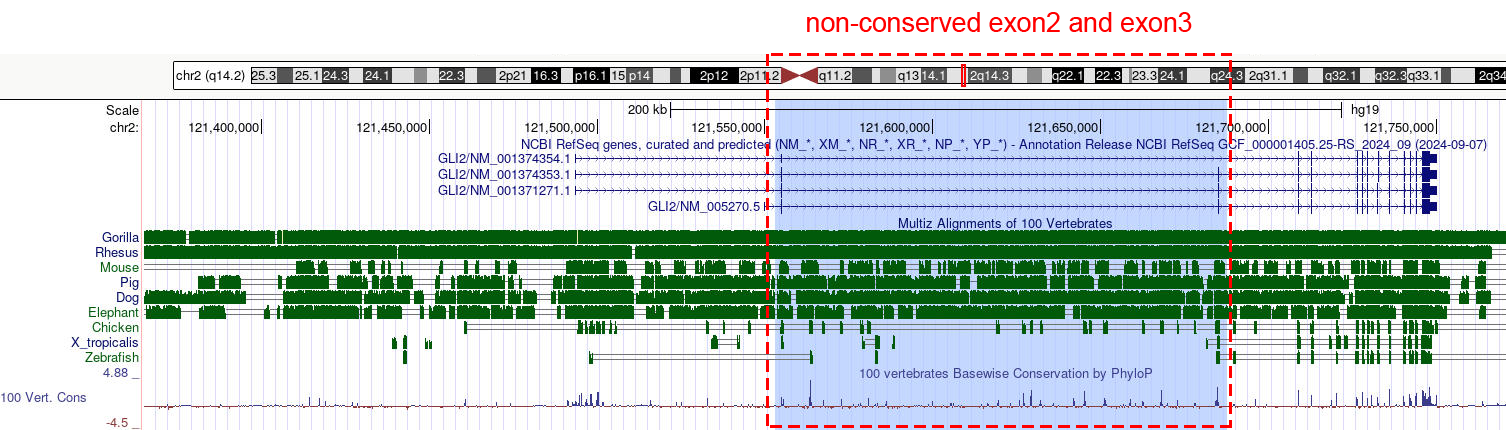

Supplement: Supplementary Figure S5 — A multi-species comparison of the non-conserved exons of the GLI2 gene. [file Image_5.tif]

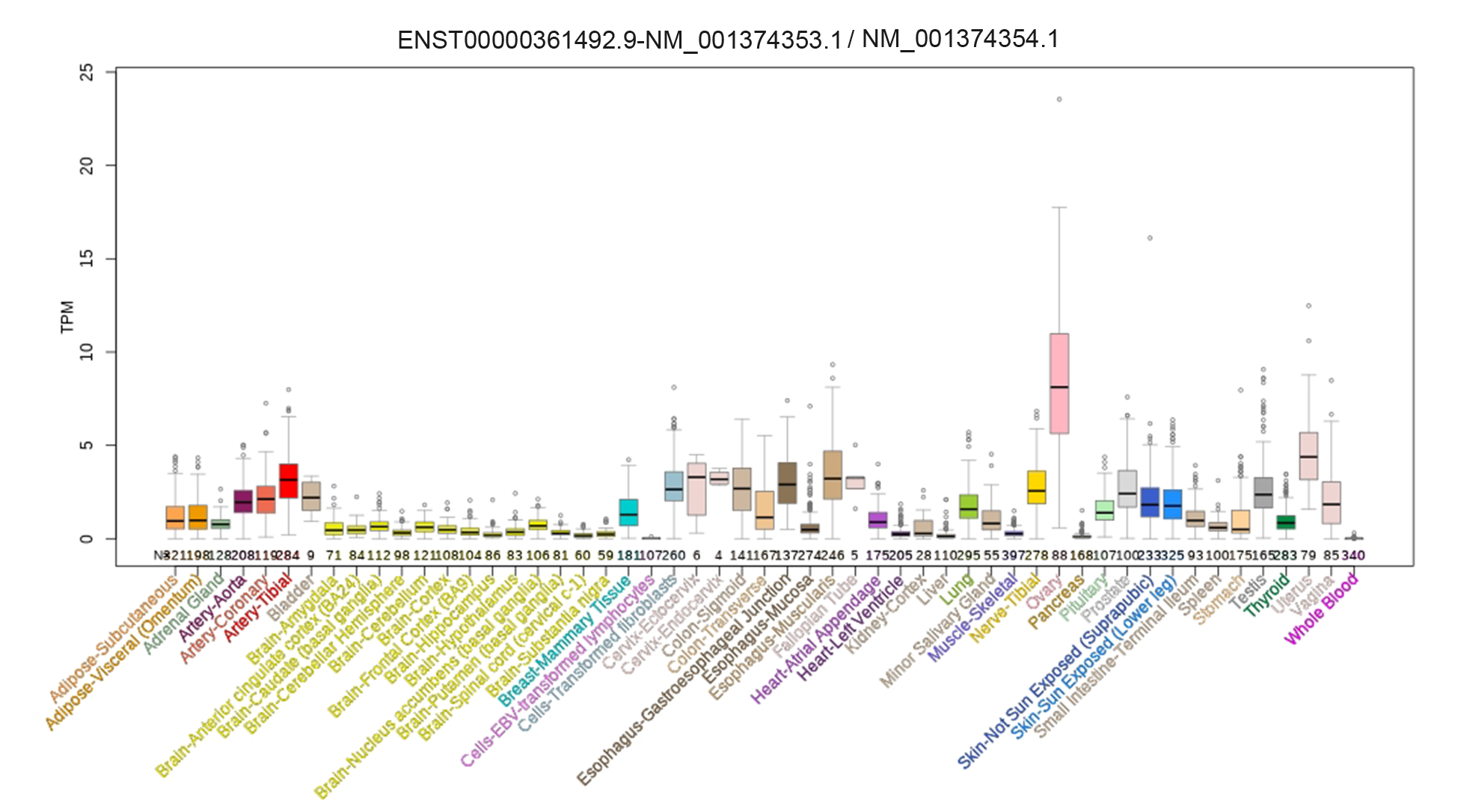

Supplement: Supplementary Figure S6 — Expression levels of the MANE transcript (NM_001374353.1) and the rescue transcript (NM_001374354.1). The MANE transcript (NM_001374353.1) and the rescue transcript (NM_001374354.1) showed comparable expression levels. [file Image_6.tif]

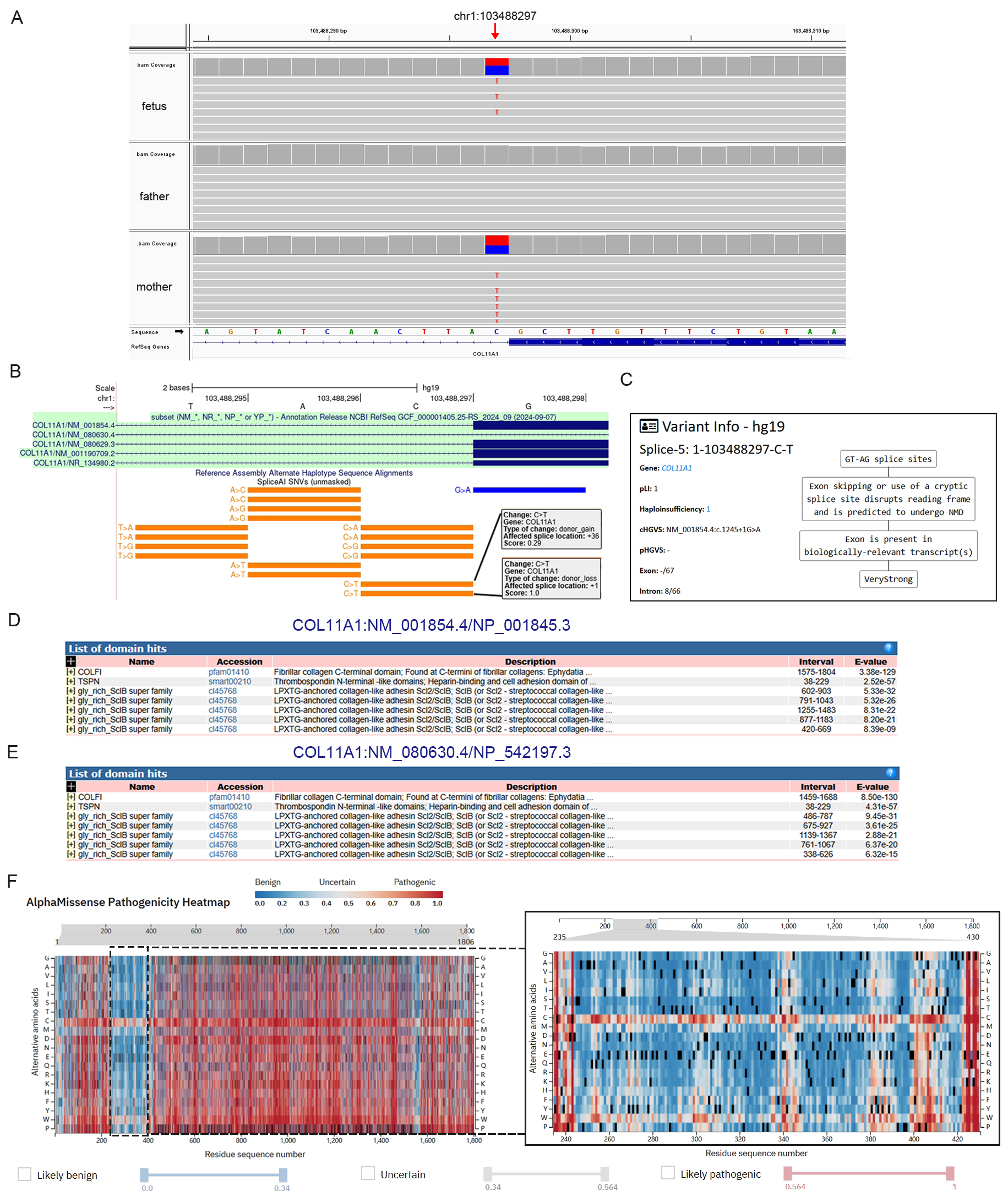

Supplement: Supplementary Figure S7 — Additional information related to the COL11A1 gene. (A) BAM file visualization of the variant. (B) SpliceAI prediction result for the variant. Black box: SpliceAI score = 0.29 (donor gain); SpliceAI score = 1.0 (donor loss). (C) AutoPVS1 prediction result for the variant. Exon skipping or use of a cryptic splice site disrupts the reading frame and is predicted to undergo NMD. ACMG classification: PVS1. (D) Domain distribution in the NP_001845.3 protein. (E) Domain distribution in the NP_542197.3 protein. (F) AlphaMissense pathogenicity heat map of the NP_001845.3 protein. Black dashed box: distribution of likely benign/VUS missense variants (243-423 aa). [file Image_7.tif]

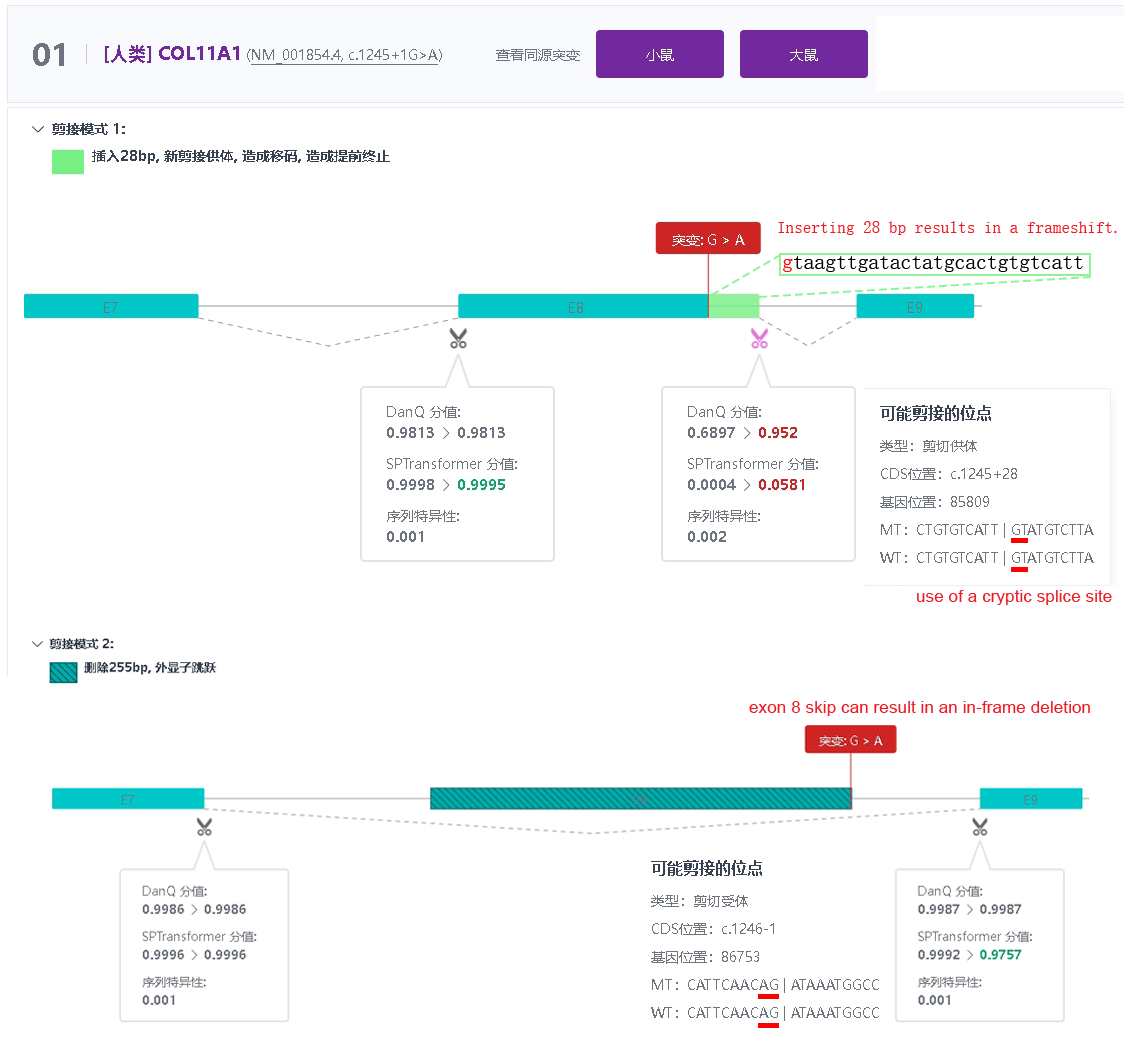

Supplement: Supplementary Figure S8 — Analysis using the RNA Splicer revealed two distinct splicing consequences: intron retention and exon 8 skipping. [file Image_8.tif]

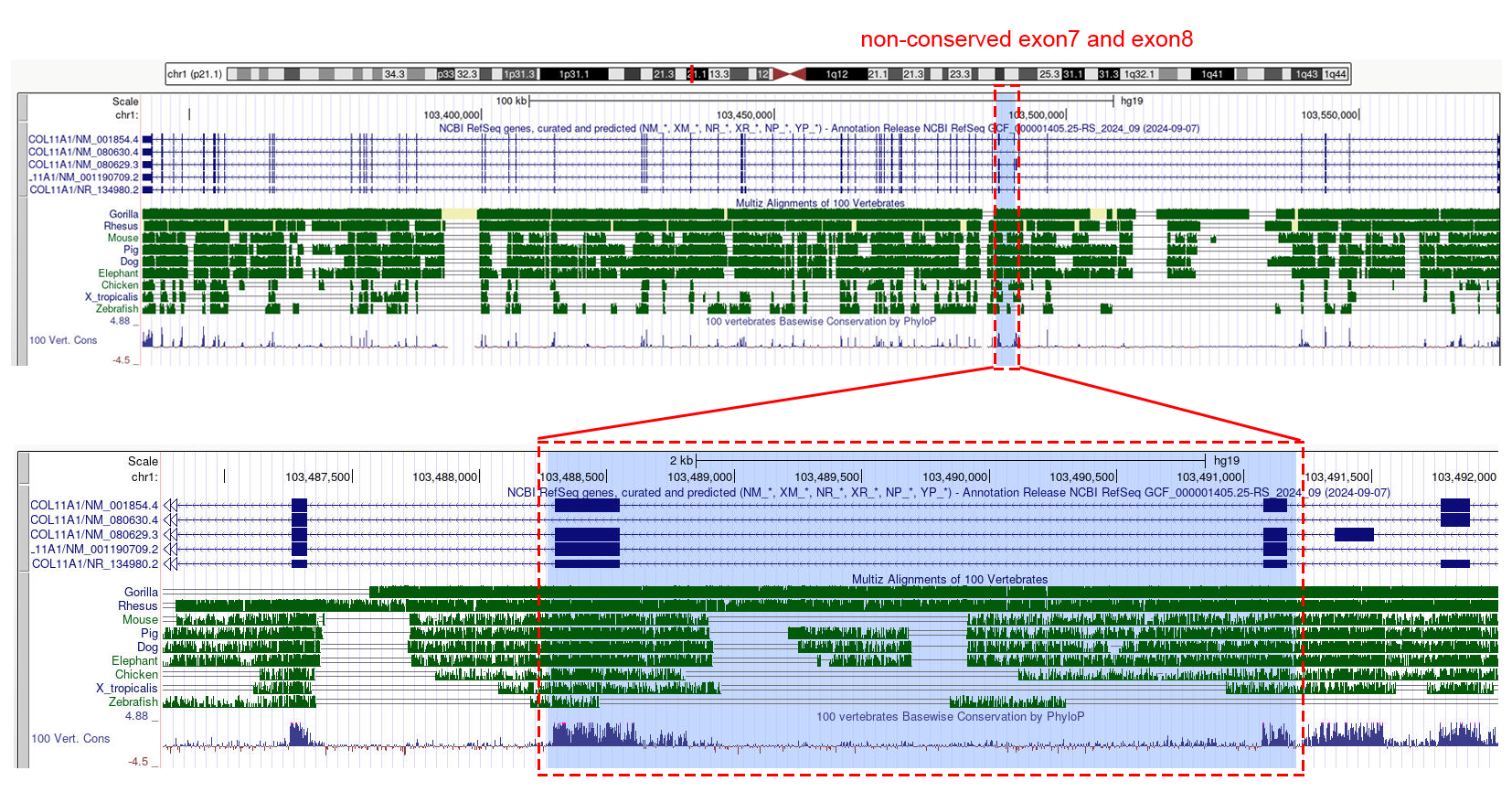

Supplement: Supplementary Figure S9 — a multi-species comparison of the non-conserved exons of the COL11A1 gene. [file Image_9.tif]

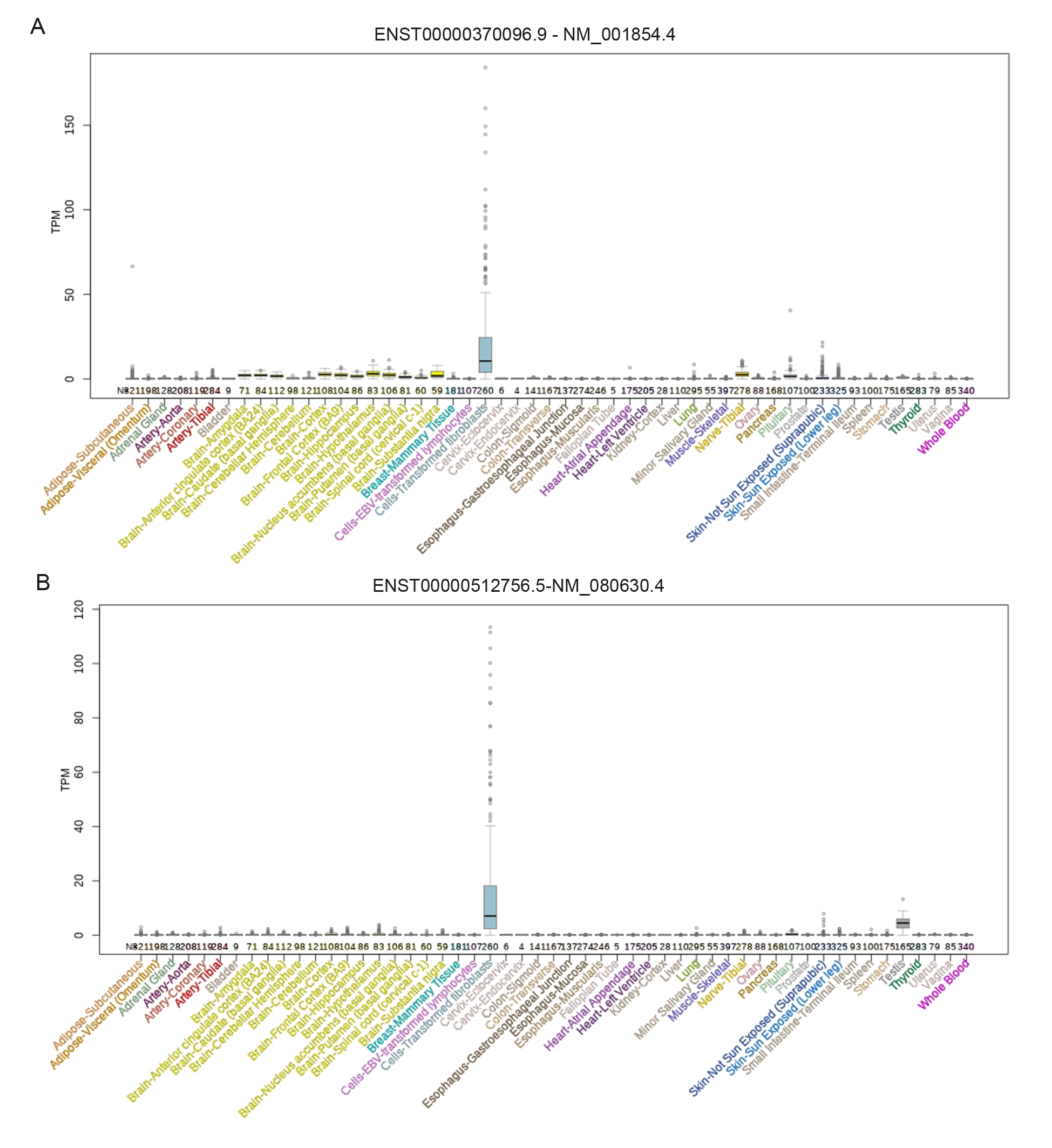

Supplement: Supplementary Figure S10 — Expression levels of the MANE transcript (NM_001854.4) and the rescue transcript (NM_080630.4). (A, B) Both the MANE transcript (NM_001854.4) and the rescue transcript (NM_080630.4) exhibited high expression specifically in cell-transformed fibroblasts. [file Image_10.tif]

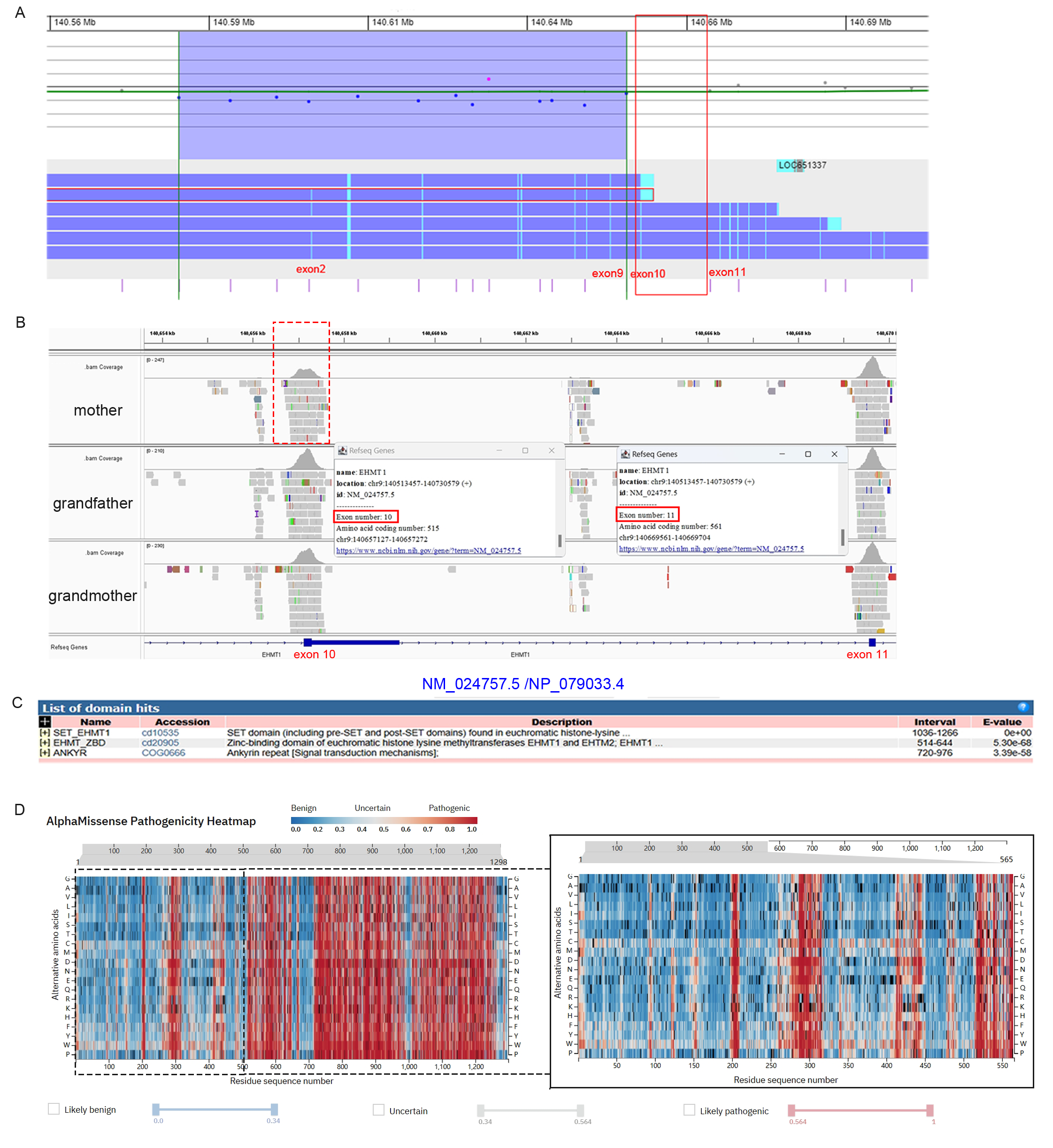

Supplement: Supplementary Figure S11 — Additional information related to the EHMT1 gene. (A) Probe distribution in CGX SNP v1.1 (Agilent, G4884A). Solid circles represent detection signals. The purple vertical line indicates preset probe positions, and the solid box indicates regions without probe coverage. (B) Reads alignment results for exons 10 and 11 of the EHMT1 gene. Red dashed box: heterozygous deletion of exon 10 in the mother. (C) Domain distribution in the NP_079033.4 protein. (D) AlphaMissense pathogenicity heat map of the NP_079033.4 protein. Black dashed box: distribution of likely benign/VUS missense variants (1-510 aa). [file Image_11.tif]

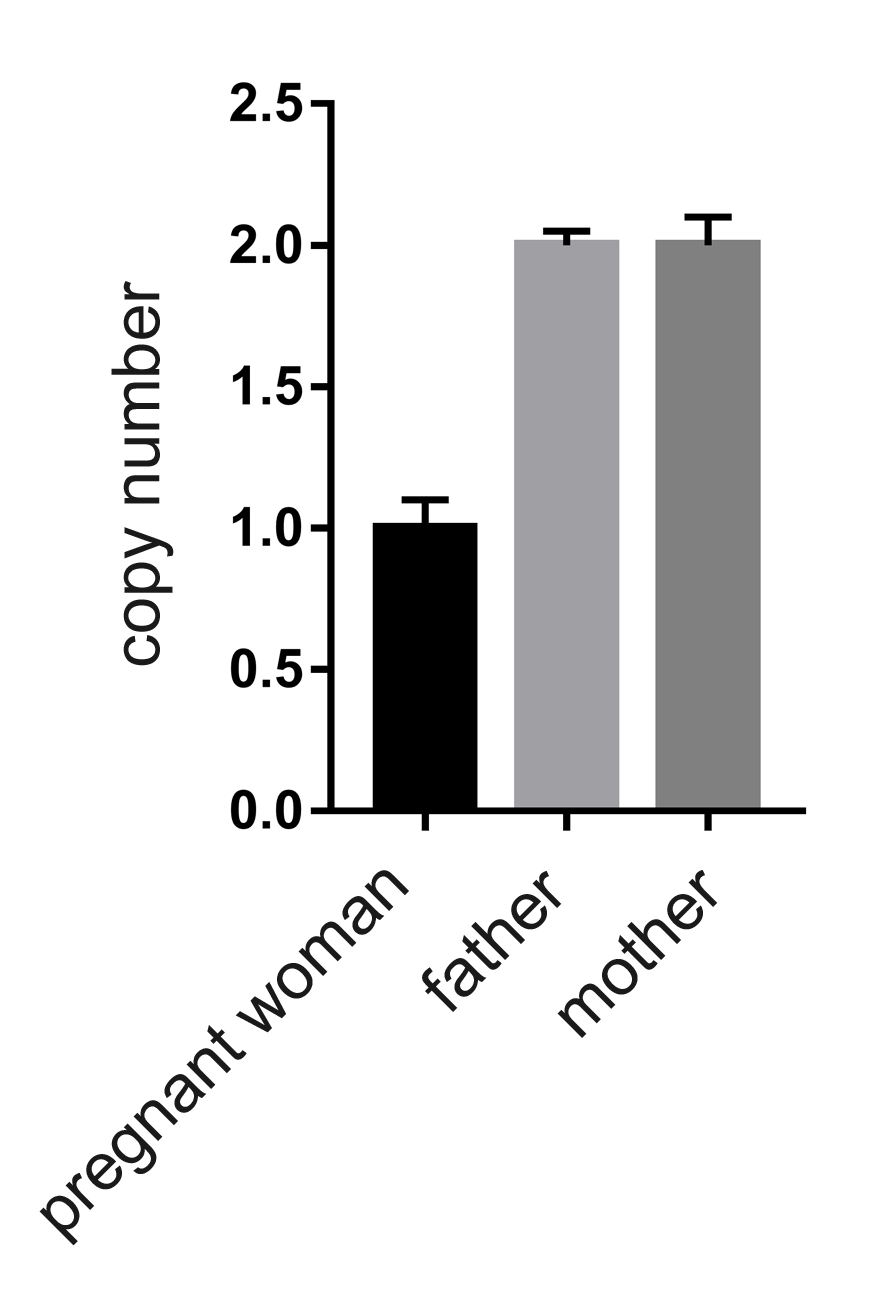

Supplement: Supplementary Figure S12 — qPCR verification of the deletion of exon 10 in the EHMT1 gene. [file Image_12.tif]

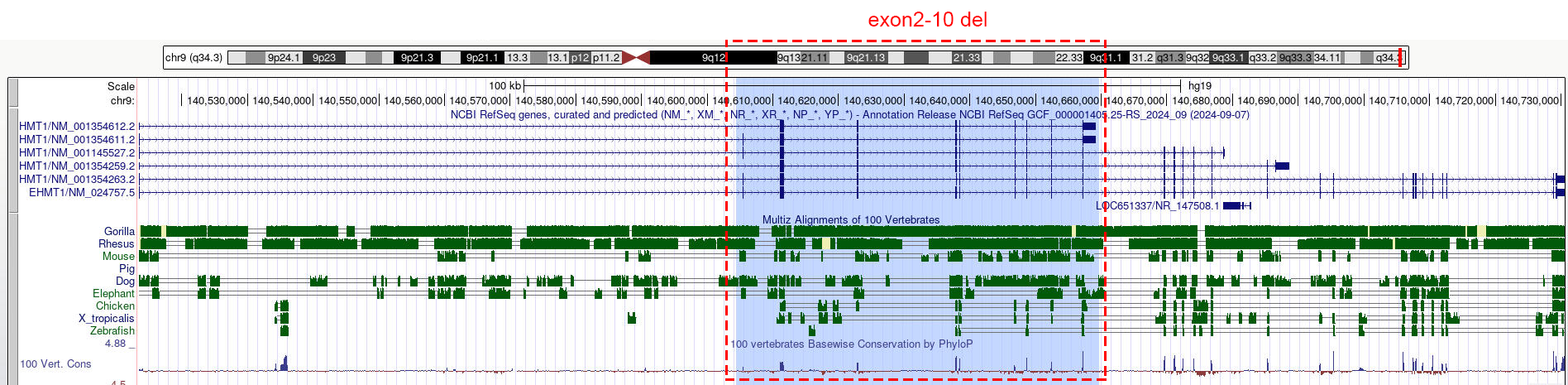

Supplement: Supplementary Figure S13 — A multi-species comparison of the non-conserved exons of the EHMT1 gene. [file Image_13.tif]
